# A hybrid architecture with bidirectional gating mechanism for spatiotemporal air quality prediction

**DOI:** 10.1038/s41598-026-46820-3

**Published:** 2026-04-08

**Authors:** Senlin Li, Bo Tang, Xiaowu Deng, Chunqiao Mi

**Affiliations:** 1 School of Computer and Artificial Intelligence, Huaihua University, Huaihua, Hunan 418008 China; 2 Hunan Provincial Key Laboratory of Intelligent Control Technology for Ecological Agriculture in Wulingshan District, Huaihua University, Huaihua, Hunan 418008 China

**Keywords:** Climate sciences, Environmental sciences, Mathematics and computing, Natural hazards

## Abstract

Accurate air quality prediction plays a crucial role in supporting various socio-economic activities, including agriculture, transportation, and disaster prevention. While traditional numerical air quality prediction methods and single-model deep learning approaches struggle to capture complex spatiotemporal dependencies, this study proposes a CNN-Transformer-LSTM model with a Bidirectional Gating Mechanism, termed BG-Hybrid, which dynamically balances local spatial features (CNN) and global temporal dependencies (Transformer) through the bidirectional gating core. The BG-Hybrid model enables adaptive feature fusion by projecting pooled features from both the two branches into sigmoid-activated weights ($\alpha , \beta$), where $\beta = 1 - \alpha$, ensuring information conservation. In terms of predictive performance, the model outperforms baseline models in error reduction and surpasses conventional hybrid architectures. This performance advantage is coupled with robust generalizability across diverse datasets, suggesting potential application in real-world scenarios.

## Introduction

Accurate air quality prediction has various socio-economic impacts on fields such as agriculture, transportation, energy management, and disaster prevention. With the intensification of climate change, the increasing frequency of extreme air quality events has made precise air quality prediction crucial for economic development and human safety^1^. According to data from the World air qualityOrganization (WMO), air quality-related disasters have increased fivefold over the past 50 years, with China being one of the most severely affected countries^2^. Taking Beijing as an example, the catastrophic “7$\cdot$21” rainstorm in 2012 resulted in 79 fatalities and economic losses exceeding 11.6 billion yuan^3^. Notably, the emissions of primary $PM_{2.5}$ from the residential and commercial sectors contribute a larger fraction to mortality in winter (57–70%) than in other seasons (28-42%).

Traditional numerical air quality prediction methods, which rely on physical models and extensive computational resources, are limited by their sensitivity to initial conditions and model parameters. Recent advances in deep learning technologies have shown great potential in capturing complex nonlinear relationships and spatiotemporal dependencies in air quality data^4,5^.

Deep learning models, particularly convolutional neural networks (CNNs) and long short-term memory networks (LSTMs), have achieved results in time series prediction tasks. CNNs can effectively extract spatial features through local convolutional operations and are widely used for spatial pattern recognition in air quality data^6^. LSTMs, known for their ability to model long-term dependencies, have shown excellent performance in time series prediction^7^. In recent years, Transformer models have demonstrated unique advantages in capturing global dependencies through self-attention mechanisms and have made breakthroughs in natural language processing and time series analysis^8,9^.

However, single-model approaches often struggle to capture the complex spatiotemporal dependencies in air quality data. For example, convolutional neural networks (CNNs) are limited in modeling long-term dependencies, while long short-term memory networks (LSTMs) are less effective in extracting spatial features. To address these limitations, researchers have begun to explore hybrid models^10^ that combine the strengths of different models to improve prediction performance. For example,^11^ proposed a CNN-LSTM Hybrid model for short-term wind speed prediction, which outperformed single-model approaches. Additionally, the combination of Transformers and CNNs has shown potential in image classification and time series prediction^12,13^.

This paper proposes a novel BG-Hybrid method with Bidirectional Gating Mechanism (abbreviated as BG-Hybrid or Hybrid), which aims to achieve high-precision air quality prediction through multi-branch feature learning and sequential modeling. Our model explicitly incorporates long-term patterns through the Transformer’s attention mechanism. Short-term patterns are captured by CNN kernels via station-specific feature maps. The gating core dynamically adjusts the weights of the Transformer and CNN.

The main contributions are as follows:

1. *Multi-branch feature extraction*^14^. The model captures global temporal dependencies and local spatial features through the CNN and Transformer module, respectively, addressing the limitations of single-model approaches in spatiotemporal dependency modeling.
2. *Dynamically gated fusion*. Bidirectional gating core^15^ dynamically adjusts the weights of the CNN and Transformer module through global average pooling and sigmoid activation, addressing the issue of feature redundancy.
3. *Experimental validation*. The models effectiveness was verified on five air quality datasets, demonstrating improvements in prediction accuracy and robustness compared to existing methods.

## Related work

Traditional air quality prediction methods, primarily based on numerical air quality prediction and statistical linear models^16^ struggle with nonlinear relationships and high-dimensional data. Recent advancements in deep neural networks^17^ have introduced data-driven approaches that leverage powerful feature extraction and modeling capabilities. Convolutional neural networks (CNNs) excel in extracting spatial features through local convolutional operations^18^, making them suitable for spatial pattern recognition in air quality data. Long short-term memory networks (LSTMs) have demonstrated excellent performance in time series prediction by modeling long-term dependencies^19^. Transformer models, with their self-attention mechanisms, excel at capturing global dependencies and have achieved breakthroughs in natural language processing and time series analysis^7^. Despite these advancements, single-model approaches still face challenges in capturing complex spatiotemporal dependencies in air quality data.

To overcome the limitations of single-model approaches, researchers have begun to explore some hybrid models that integrate the strengths of different architectures to enhance prediction performance. For example, a CNN-LSTM hybrid model^20^ was proposed for short-term typhoon prediction, which outperformed single-model approaches. Additionally, the combination of Transformer and CNN has shown great potential in image classification and time series prediction^21^. These studies have demonstrated that some hybrid models can effectively integrate multi-scale features, thereby improving prediction accuracy and robustness^22^. Multimodal data fusion is another important direction for improving the accuracy of air quality prediction.^23^ proposed a multimodal fusion network that combines CNN and Transformer modules, significantly enhancing the accuracy of precipitation prediction. These advancements indicate that Hybrid models and multimodal data fusion are effective approaches for addressing complex air quality prediction problems. However, pure Transformer architectures often struggle with local spatial feature extraction, particularly for high-resolution grid data.

## Methodology

### Model architecture

**Fig. 1:**
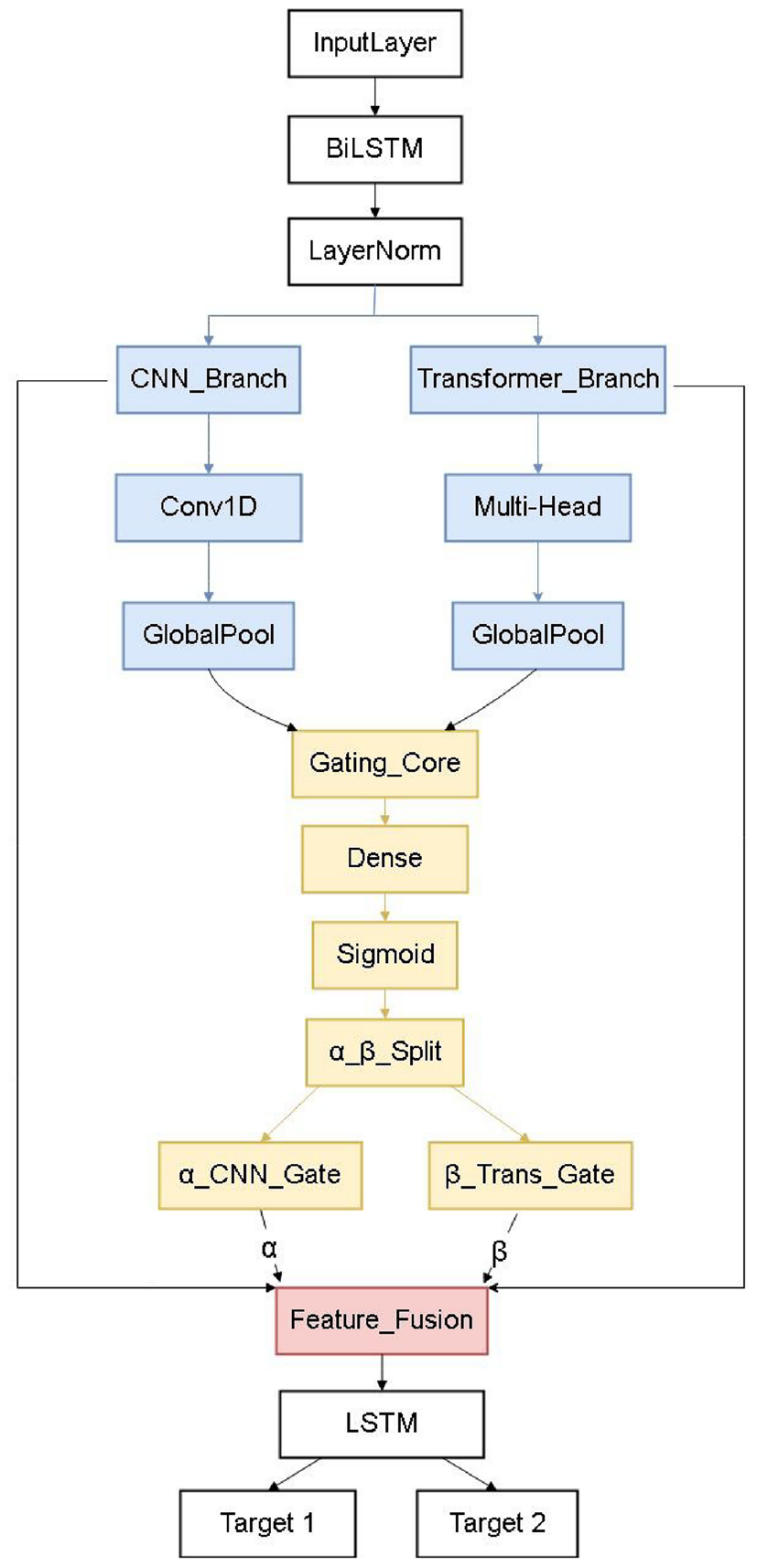
The architecture of the Hybrid model. At the bottom layer, the bidirectional long short-term memory network (Bi-LSTM) extracts temporal dynamic features from time slices ; in the middle layer, the Transformer module composed of multi-head attention (Multi-Head Attention) and one-dimensional convolution (Conv1D) is utilized to obtain global temporal and local spatial features; Subsequently, the feature fusion module integrates the features, and then the long short-term memory network (LSTM) processes the temporal dynamics, and finally, the fully-connected layer (Dense) outputs the results.

The BG-Hybrid or Hybrid method proposed in this study aims to achieve high-precision air quality prediction through multi-branch feature learning and sequential modeling. The overall architecture of the model is shown in Figure 1, which consists of three main components: a Bi-LSTM for temporal feature extraction, parallel CNN and Transformer branches for spatial and global temporal feature extraction, and a bidirectional gating core (BGC) for adaptive fusion.

The main functions of the modules are as follows:

**Spatial feature extraction**

Conv1D Convolutional Layer: After every time slice input data (time_sequence*features), has its preliminary temporal features extracted by the Bi-LSTM, it enters the Transformer and Conv1D layers^24^. The convolution kernel of the Conv1D slides along the feature dimension of the time series data. Through the convolution operation, it extracts local spatial features. For example, it can capture the local correlation features between data points on adjacent time slices. After convolution, the obtained data have a dimension of (8*128), which increases the richness of the data features.

The process of data dimension transformation is as follows: the input data is configured with 16 time steps, each containing 9 features. The nine input variables are: PM$\vphantom{0}_{2.5}$, PM$\vphantom{0}_{10}$, SO$\vphantom{0}_{2}$, NO$\vphantom{0}_{2}$, CO, O$\vphantom{0}_{3}$, temperature, relative humidity, and wind speed. First, it is processed by a Bidirectional LSTM, where each direction is assigned 87 hidden units (determined through parameter optimization). After bidirectional concatenation, the feature dimension of each time step becomes 174, resulting in a representation of 16 time steps $\times$ 174 dimensions. The data then enters the Conv1D layer, which slides a convolutional kernel along the time dimension, compressing the 174-dimensional features of each time step down to 82 dimensions while keeping the number of time steps at 16 (yielding 16 time steps $\times$ 82 dimensions). Next, MaxPooling1D with a pool size of 2 is applied to downsample the time steps, reducing their number from 16 to 8, while the feature dimension remains at 82 (resulting in 8 time steps $\times$ 82 dimensions). Finally, a Dense(128) layer is used to perform a fully connected mapping on each time step’s 82-dimensional vector, elevating it to 128 dimensions, ultimately forming a feature representation of 8 time steps, each with 128 dimensions – that is, a shape of (8, 128).

Transformer Module: In the BG-Hybrid architecture, the Transformer module^25^ is not a standard implementation but a tailored design to address air quality data’s long-range temporal dependency challenge, complementing the CNN module’s local spatial extraction capability. Our key improvements lie in two aspects: first, we optimize the self-attention window to adapt to air qualitytime-series characteristics–constraining the attention scope within a sliding time window (16 consecutive time steps) to reduce computational complexity while retaining effective global correlations; second, we introduce a feature alignment layer to unify the semantic space of Transformer-extracted temporal features and CNN-extracted spatial features, ensuring seamless fusion in the subsequent bidirectional gating mechanism (BGC). This tailored design maintains consistent output dimensions (16$\times$128) for compatibility. To match the temporal dimension of the CNN branch, a MaxPooling1D(pool_size=2) is applied to the Transformer features, yielding shape (8, 128) before fusion.

**Temporal feature extraction**

Bi-LSTM Layer: The underlying input data passes through the Bi-LSTM (Bidirectional Long Short-Term Memory Network) layer. The Bi-LSTM processes the time series data in both forward and backward directions^26^, which enables it to better capture the forward and backward dependencies of the time series and effectively extract the dynamic features in the temporal dimension. The output dimension is (16, 174), retaining the temporal dynamic information of the time series.

LSTM Layer: After the feature fusion (feature fusion), the data enters the LSTM layer. The LSTM layer further processes the time series data. Through its gating mechanisms (forget gate, input gate, output gate), it can selectively remember or forget the information in the time series, capture the long-term temporal dependencies, and dig deeper into the dynamic features in the temporal dimension.

**Gating mechanism and feature fusion**

To address feature redundancy in parallel CNN-Transformer module, we propose a bidirectional gating core (BGC) (see Figure 1), which dynamically balances local (CNN) and global (Transformer) features via learnable weights.

The BGC operates is as follows:

Global average pooling (GAP) is applied to both branches’ outputs. The pooled features are concatenated and projected through a fully connected (FC) layer with sigmoid activation to produce the gating weight $\alpha$:

1 $$\begin{aligned} \alpha = \sigma (W_g \cdot \text {Concat}(F_{\text {CNN}}^{\text {pool}}, F_{\text {Trans}}^{\text {pool}}) + b_g). \end{aligned}$$

Then, the final fused feature is computed as:

2 $$\begin{aligned} F_{\text {fused}} = \alpha \cdot \text {Proj}(F_{\text {CNN}}) + \beta \cdot F_{\text {Trans}}. \end{aligned}$$

Here, $\text {Proj}(\cdot )$ aligns the CNN features’ dimension with the Transformer’s output. The gating weights $\alpha$ and $\beta$ are learned adaptively during training, allowing the model to emphasize CNN-extracted local features when short-term patterns dominate, and shift focus to Transformer-captured global dependencies when long-range trends are more relevant.

**Transformer**

The Transformer module captures global temporal dependencies in the input sequence through the self-attention mechanism. The self-attention computes the correlations between each time step in the sequence, effectively modeling long-range dependencies. Initially, the input data $X \in \mathbb {R}^{T \times D}$ is transformed into a higher-dimensional representation $E \in \mathbb {R}^{T \times d_{\text {model}}}$ through an embedding layer, where *T* represents the number of time steps, *D* is the input feature dimension, and $d_{\text {model}}$ is the embedding dimension. Within the module, self-attention captures global dependencies by computing the correlations between *Queries* (*Q*), *Keys* (*K*), and *Values* (*V*):

3 $$\begin{aligned} Q = EW_Q, \quad K = EW_K, \quad V = EW_V. \end{aligned}$$

where, $W_Q$, $W_K$, and $W_V$ are learnable weight matrices.

The formula for calculating attention scores is:

4 $$\begin{aligned} \text {Attention}(Q, K, V) = \text {softmax}\left( \frac{QK^T}{\sqrt{d_k}}\right) V. \end{aligned}$$

where, $d_k$ is the dimension of the key vectors, which scales the attention scores to prevent gradient vanishing, and the softmax function normalizes the attention weights.

The *Multi-Head Attention* mechanism shown in Algorithm 1 enhances the model’s representational capability through parallel attention heads:

5 $$\begin{aligned} \text {MultiHead}(Q,K,V) = \text {Concat}(\text {head}_1, ..., \text {head}_h)W^O, \end{aligned}$$

where, each head is computed as:

6 $$\begin{aligned} \text {head}_i = \text {Attention}(QW_i^Q, KW_i^K, VW_i^V), \end{aligned}$$

where, $W_i$ represents the weight matrix for the i-th head.

**Algorithm 1:**
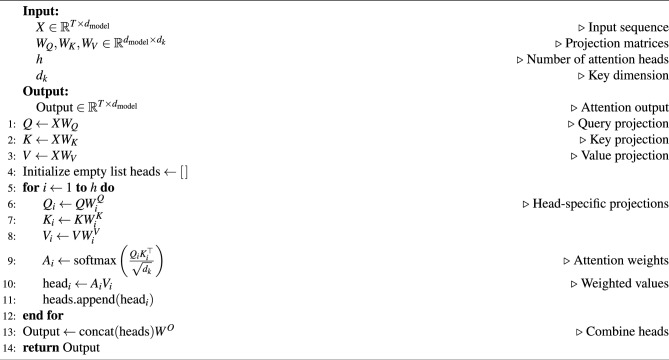
Multi-head attention mechanism

The Multi-Head Attention output is further processed through a feedforward network (FFN) to extract additional features:

7 $$\begin{aligned} \text {FFN}(x) = \max (0, xW_1 + b_1)W_2 + b_2, \end{aligned}$$

where, $W_1$ and $W_2$ are the weight matrices, and $b_1$, $b_2$ are the bias terms. The final output is passed through a Flatten layer to facilitate subsequent feature fusion.

## CNN module

The CNN module extracts local spatial features through convolutional operations. Convolutional Neural Networks (CNNs) have demonstrated excellent performance in spatial pattern recognition of air quality data, effectively capturing local spatial dependencies^27^.

For input data $X \in \mathbb {R}^{T \times D}$, the 1D convolutional operation (Conv1D) is defined as:

8 $$\begin{aligned} \text {Conv1D}(X) = \sigma (W_c *X + b_c), \end{aligned}$$

where:

- $W_c$ represents the convolutional kernel weights.
- $b_c$ is the bias term.
- $*$ denotes the convolution operation.
- $\sigma$ is the ReLU activation function ($\sigma (z) = \max (0, z)$).

The convolutional layer output is subsequently downsampled through a MaxPooling1D layer.

**LSTM**

The features from the Transformer and CNN modules are fed into a fusion layer to form a multi-scale feature representation. The fused features are then processed by the LSTM module for sequential modeling to capture dynamic changes^28^. Then, the feature fusion module^29^ combines the outputs from both the Transformer and CNN modules see Equation 2.

The fused features are then processed by an LSTM layer:

9 $$\begin{aligned} h_t, c_t = \text {LSTM}(E^{\text {fused}}, h_{t-1}, c_{t-1}), \end{aligned}$$

where, $h_t$ is the hidden state at time *t*, and $c_t$ is the cell state at time *t*.

The output of the LSTM module is passed through a fully connected layer (FC) for nonlinear mapping to produce the final prediction. The fully connected layer effectively integrates high-level features, thereby enhancing the model’s prediction accuracy. The specific implementation is as follows:

Fully Connected Layer: The output of the LSTM is processed through the fully connected layer for nonlinear mapping: The fully connected layer applies a nonlinear transformation:

10 $$\begin{aligned} y = \sigma (W_f H_c + b_f), \end{aligned}$$

where, $W_f$ is the weight matrix of the fully connected layer, and $b_f$ is the corresponding bias term.

### Output layer

The final prediction is obtained through a linear transformation:

11 $$\begin{aligned} \hat{y} = W_o y + b_o, \end{aligned}$$

where, $W_o$ is the output weight matrix, and $b_o$ is the output bias term.

### Implementation details

To optimize model parameters, we performed Bayesian hyperparameter optimization ( Algorithm 2) and finally determined the hyperparameter configuration of the Hybrid model. The parameters for the Transformer module are adjusted as follows: the number of attention heads (num_heads) is set to 4, which splits the input data into multiple subspaces, enabling the capture of temporal dependencies across different dimensions. The dimension of the feedforward network (ff_dim) is set to 64, which serves to enhance the feature representation capabilities and further improve the model’s ability to capture features through nonlinear transformations. Additionally, a dropout rate (dropout_rate) of 0.1 is configured to randomly discard the outputs of some neurons during the training process, thereby preventing overfitting.

**Algorithm 2:**
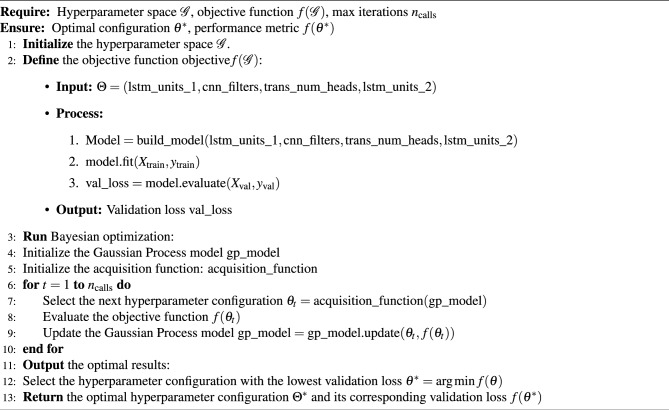
Bayesian hyperparameter optimization

For the CNN module, the parameters are configured as follows: the number of convolutional filters (cnn_filters) is set to 82 to extract different local features. The kernel size (kernel_size) is 3$\times$1, indicating that the convolutional kernel covers 3 time steps in the temporal dimension and 1 unit in the spatial dimension, and is used to capture local patterns. The pooling window size (pool_size) for the pooling layer is 2, which is employed to reduce the dimensionality of the feature maps while enhancing the robustness of the features.

The LSTM module, a recurrent neural network structure, is specifically designed to model the dynamic changes in time series data. In this optimized model, the number of Bi-LSTM units for the first layer (lstm_units) is set to 87, and for LSTM, it is set to 256. These settings mean there are 87 neurons in the first LSTM layer and 256 neurons in the second LSTM layer, which are capable of capturing long-term dependencies in the time series data more effectively .Furthermore, the optimal learning rate is set to 0.001, the optimal window size is 16, the optimal prediction step is 3, and the optimal batch size is 64. These parameter values contribute to the overall performance improvement of the Hybrid model. During training, the model adopts the Adam optimizer with an initial learning rate (lr) of 0.001 to adjust the model parameters and minimize the loss function. Additionally, the dataset is divided into training, validation, and test sets with a ratio of 6:2:2. The training set is used for model training, the validation set for monitoring overfitting, and the test set for evaluating the models performance. By assessing the models performance on the validation set, we adjust model parameters and training strategies in a timely manner to ensure the model maintains strong generalization capabilities during training.

### Metrics

To quantify the model performance, we adopted the following evaluation metrics:

12 $$\begin{aligned} \textrm{RMSE}= & \sqrt{\frac{1}{N} \sum _{i=1}^{N} (y_i - \hat{y}_i)^2} \end{aligned}$$

13 $$\begin{aligned} \textrm{MAE}= & \frac{1}{N} \sum _{i=1}^{N} |y_i - \hat{y}_i| \end{aligned}$$

14 $$\begin{aligned} R^2= & 1 - \frac{\displaystyle \sum _{i=1}^{N} (y_i - \hat{y}_i)^2}{\displaystyle \sum _{i=1}^{N} (y_i - \overline{y})^2}. \end{aligned}$$

where, $N$ is the sample size, $\hat{y}_i$ is the predicted value, and $y_i$ is the true value.

## Experiments

### Data

To verify the effectiveness of the proposed BG-Hybrid model, we conducted extensive experiments on five air quality datasets from distinct geographical regions shown in Table 1.

**Table 1:** Statistical information of all dataset.

Dataset	Location	Coordinates	Elevation	Temporal
		(Lat; Lon)	(m)	Resolution
Aoti	Beijing, China	39.992; 116.463	45	Hourly
Huairou	Beijing, China	40.315; 116.632	100	Hourly
Wanliu	Beijing, China	39.987; 116.290	50	Hourly
Wulingshan	Hunan, China	27.950; 110.717	1500	Daily
Jena Climate	Thuringia, Germany	50.927; 11.586	160	10-min

All datasets were selected based on: (1) Data completeness ($\ge$95% valid records), (2) Full seasonal coverage (all four seasons), (3) Representative geographical distribution (urban, suburban, and mountainous areas). We evaluate the model on two prediction targets.

### Baseline models

To comprehensively evaluate the performance of the proposed model in this study, we compared it with the following methods:

- LSTM controls the flow of information through the input gate, forget gate, and output gate, and it can effectively handle the long-term dependencies in long sequential data^30^.
- CNN mainly uses components such as convolutional layers, pooling layers, and fully connected layers to automatically extract the features of data. In time series prediction, the temporal convolutional network applies the convolution operation to time series data. By sliding the convolution kernel along the time dimension, it extracts local features and can capture the short-term patterns and trends in the data.
- GRU simplifies the structure of LSTM by combining the input gate, forget gate, and output gate into an update gate and a reset gate^30^. Through these two gates, it controls the flow of information and decides which information needs to be retained and updated. It has a good ability to model short-term and medium-term dependencies.
- CNN_LSTM combines the advantages of CNN and LSTM. Firstly, it uses the convolutional layers of CNN to capture the local spatial features and short-term temporal features in the data. Then, it utilizes the memory units of LSTM to handle the long-term temporal dependencies and further excavate the long-term patterns and trends in the data.
- NO_CNN (a Hybrid model combining Transformer and LSTM): This model retains all components of the BG-Hybrid architecture except the CNN branch. It leverages the Transformer for global temporal dependency capture and LSTM for sequential modeling, maintaining other core modules such as Bi-LSTM and the bidirectional gating core (BGC) to ensure consistency with the original framework.^31^.
- NO_TRANSFORMER (a Hybrid model for extracting spatiotemporal features): This model retains all components of the BG-Hybrid architecture except the Transformer branch. It utilizes the CNN for local spatial feature extraction and LSTM for sequential modeling, preserving Bi-LSTM and BGC to avoid structural deviations from the original framework, while reducing computational complexity associated with the Transformer..

## Result

### Performance metrics comparison

Table 2 presents the absolute predictive metrics of the BG-Hybrid model and baseline models across datasets 5 times.

**Table 2:** Absolute average performance of each prediction model (header split into two lines for compactness).

Model	MAE (mean ± std)		RMSE (mean ± std)		$R^2$ (mean ± std)	
	AQI	PM$\vphantom{0}_{2.5}$ ($\mu \textrm{g}/\textrm{m}^3$)	AQI	PM$\vphantom{0}_{2.5}$ ($\mu \textrm{g}/\textrm{m}^3$)	AQI	PM$\vphantom{0}_{2.5}$ ($\mu \textrm{g}/\textrm{m}^3$)
BG-Hybrid	3.12 ± 0.25	6.85 ± 0.57	4.78 ± 0.86	9.92 ± 1.24	0.891 ± 0.018	0.873 ± 0.022
CNN_LSTM	3.77 ± 0.37	8.19 ± 0.73	5.55 ± 0.84	11.52 ± 1.21	0.863 ± 0.020	0.845 ± 0.024
GRU	3.96 ± 0.29	8.64 ± 0.61	5.82 ± 0.85	11.98 ± 1.22	0.850 ± 0.023	0.832 ± 0.026
LSTM	4.06 ± 0.34	8.83 ± 0.70	5.93 ± 0.81	12.17 ± 1.18	0.847 ± 0.022	0.829 ± 0.025
NO_TRANSFORMER	4.33 ± 0.44	9.38 ± 0.91	6.16 ± 0.90	12.59 ± 1.31	0.835 ± 0.025	0.817 ± 0.029
NO_CNN	4.64 ± 0.34	10.05 ± 0.70	6.48 ± 0.80	13.25 ± 1.17	0.794 ± 0.027	0.775 ± 0.031
CNN	4.77 ± 0.40	10.32 ± 0.82	6.62 ± 0.87	13.49 ± 1.26	0.804 ± 0.024	0.784 ± 0.028

Key results include:

1. *Error reduction* The BG-Hybrid model achieves an average improvement of 20-35% in MAE and RMSE compared to single models (CNN/LSTM), and 12-20% compared to CNN-LSTM.
2. *Improved goodness-of-fit:* The R^2^ score increases by an average of 3-12%, indicating its superior ability to capture underlying data patterns.
3. *Dual-target consistency:* It delivers optimal performance for both prediction targets, demonstrating strong generalization capability.

The BG-Hybrid model integrates the strengths of all three components: it employs Transformer to supplement LSTM’s global temporal dependency modeling capability, and utilizes CNN to enhance Transformer’s local spatial feature extraction capability, and leverages CNN to compress Transformer’s local information for reduced computational complexity. This achieves efficient collaborative modeling of spatiotemporal features.

Additionally, the CNN_LSTM model performed second best to the BG-Hybrid model in MAE and RMSE, and also achieved a high R^2^. This suggests that it has good predictive accuracy and error control. The CNN_LSTM model combines CNN and LSTM, using CNN to extract local features and LSTM to model time series, a structure that performs well when dealing with data that has certain spatiotemporal correlations.

**Fig. 2:**
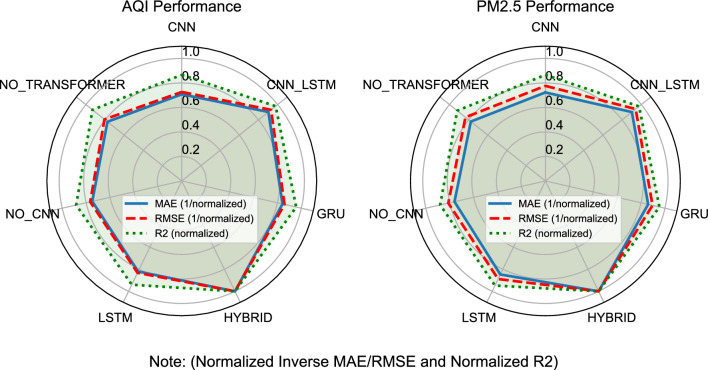
Performance comparison of different models. The closer the value is to the edge of the radar chart, the better the model performs under the corresponding metric. AQI (left) and PM$\vphantom{0}_{2.5}$ ($\mu \textrm{g}/\textrm{m}^3$) (right) results are normalized separately to [0,1] for visualization; absolute metrics are reported in Table 2.

Figure 2 also shows that the BG-Hybrid model performs the best when considering both error and fit comprehensively. The predictive metric data, for each model has been normalized, with values closer to 1 being better. Normalization allows different metrics to be compared on the same scale, which is very useful for directly comparing the performance of different models. Note that AQI and PM$\vphantom{0}_{2.5}$ ($\mu \textrm{g}/\textrm{m}^3$) are normalized independently and plotted on the same radar chart only for visual comparison.

**Fig. 3:**
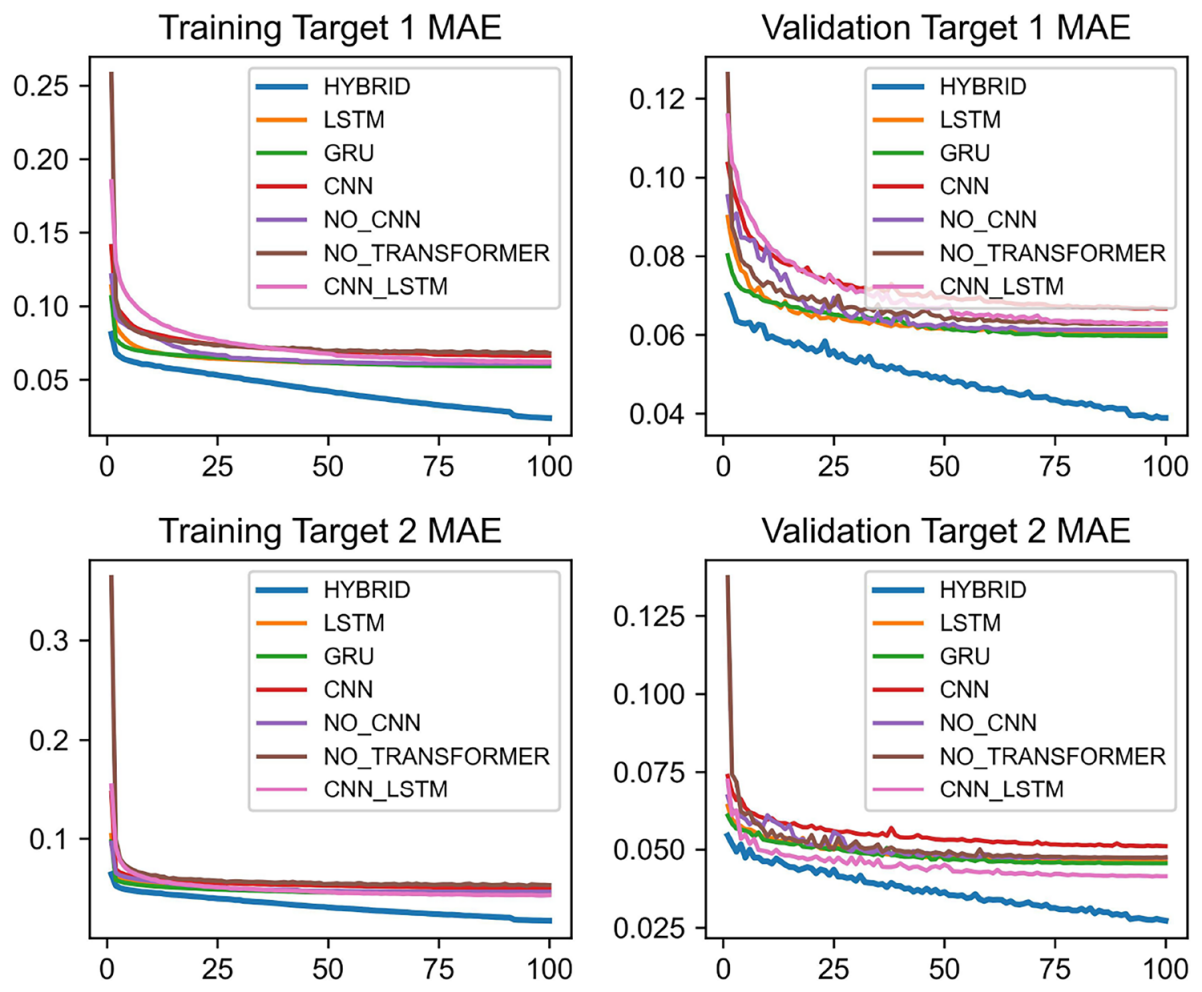
Changes in mean absolute error (MAE) during training and validation for different models.

**Fig. 4:**
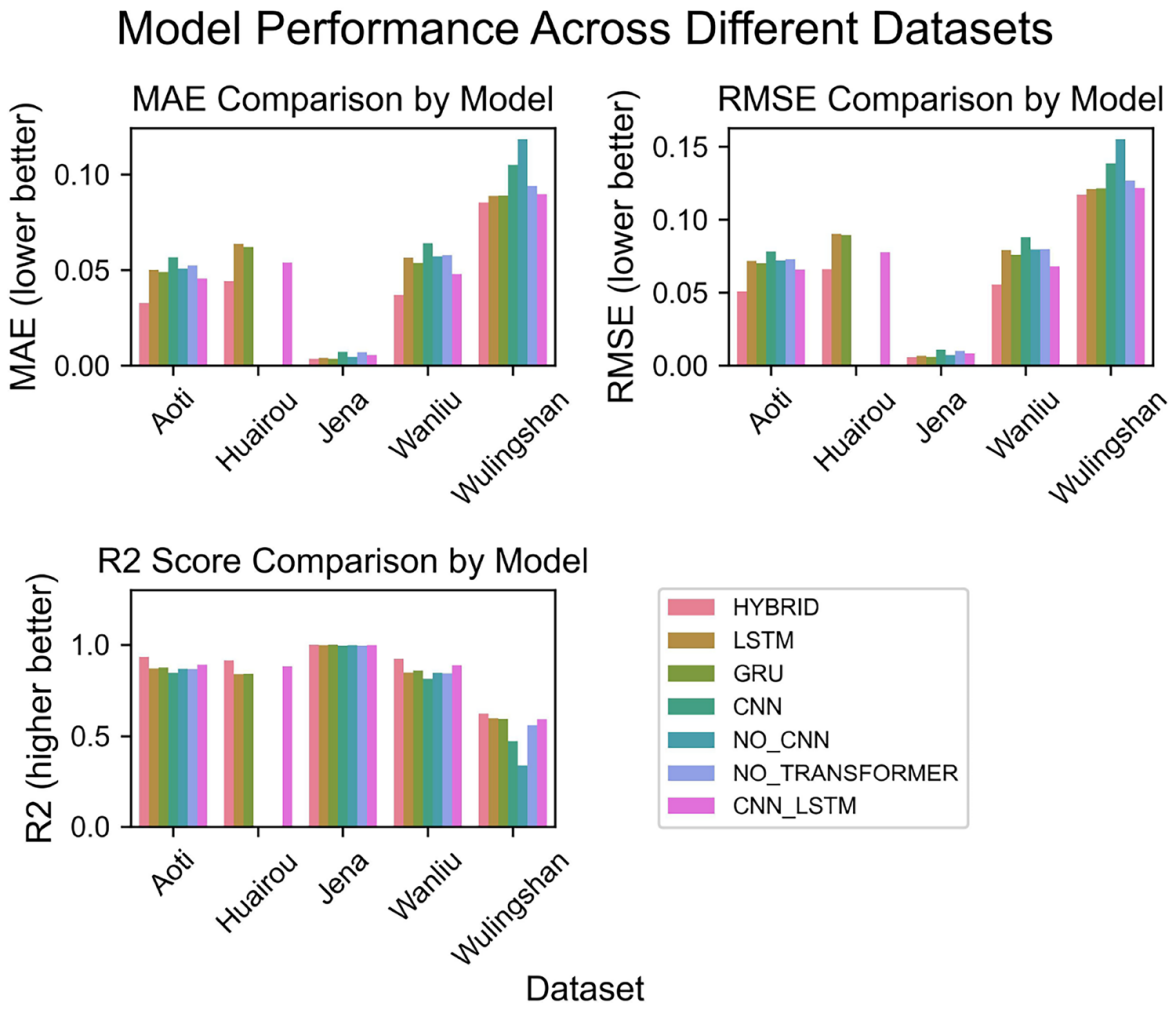
Detailed performance comparison of different models across multiple datasets.

Figure 3 shows the changes in the average training loss and average validation loss of each model across all datasets. The BG-Hybrid model demonstrates consistently low Mean Absolute Error (MAE) for both Target 1 (*AQI*) and Target 2 ($PM_{2.5}$) on the training and validation sets, indicating strong fitting and generalization capabilities. This might be because the BG-Hybrid model integrates the powerful global and local feature extraction capabilities of Transformer and CNN, as well as the advantage of LSTM in capturing long-term dependencies when handling sequential data. Through complementary advantages, it can more comprehensively process complex data and improve the overall performance. It can flexibly adjust the weights or structures of its components, better adapt to diverse datasets and application scenarios, effectively reduce the risk of overfitting, and enhance generalization ability. The CNN_LSTM model performs well in the indicators related to Target 1 (AQI), while the LSTM model performs well in the indicators related to Target 2 ($PM_{2.5}$). In contrast, the NO_CNN and NO_TRANSFORMER models have relatively high MAE values for both targets on both the training set and the validation set, and their performance is weaker among these compared models.

In terms of computational efficiency, the BG-Hybrid model–having the largest number of parameters–trains longer than the baseline models. This extra cost is justified by performance gains, and Bayesian hyper-parameter optimization keeps the model practical by eliminating redundant training runs.

### Robustness analysis

#### Comparison of comprehensive performance across datasets

Figure 4 provides a detailed comparison of Mean Absolute Error (MAE), Root Mean Square Error (RMSE), and R^2^ score of multiple models on different datasets (Aoti, Huairou, Jena, Wanliu, Wulingshan). In the MAE and RMSE metrics, a lower value indicates a smaller prediction error of the model. The BG-Hybrid model has lower MAE and RMSE values on most datasets, and the fluctuation range is smaller than that of some single models (such as LSTM, GRU), indicating that it can maintain a smaller prediction error under different data characteristics and has strong adaptability to datasets. The R^2^ score of the BG-Hybrid on each dataset is generally high (close to 1), and its stability is better than that of some comparison models (such as CNN, NO_CNN), indicating that it can effectively capture the potential patterns of different datasets and has stable fitting performance. These data show that the BG-Hybrid model enhances adaptability to different datasets by integrating the characteristics of multiple models (such as capturing local features and handling sequence dependencies). For example, on the Huairou dataset, the MAE and RMSE of BG-Hybrid are significantly lower than those of models like LSTM and GRU, and the R^2^ score is higher, reflecting its stability under complex data characteristics. This robustness makes it suitable for diverse practical scenarios, thereby reducing the risk of performance fluctuations due to differences in data distribution. However, although BG-Hybrid has strong overall robustness, on individual datasets (such as Wulingshan), its RMSE is not the lowest, indicating that under specific data distributions, a single model (such as CNN_LSTM) may perform better in a certain indicator. Furthermore, the structural complexity of the BG-Hybrid model may lead to an increase in training costs (time, computational resources), so it is necessary to balance performance and efficiency in practical applications.

**Fig. 5:**
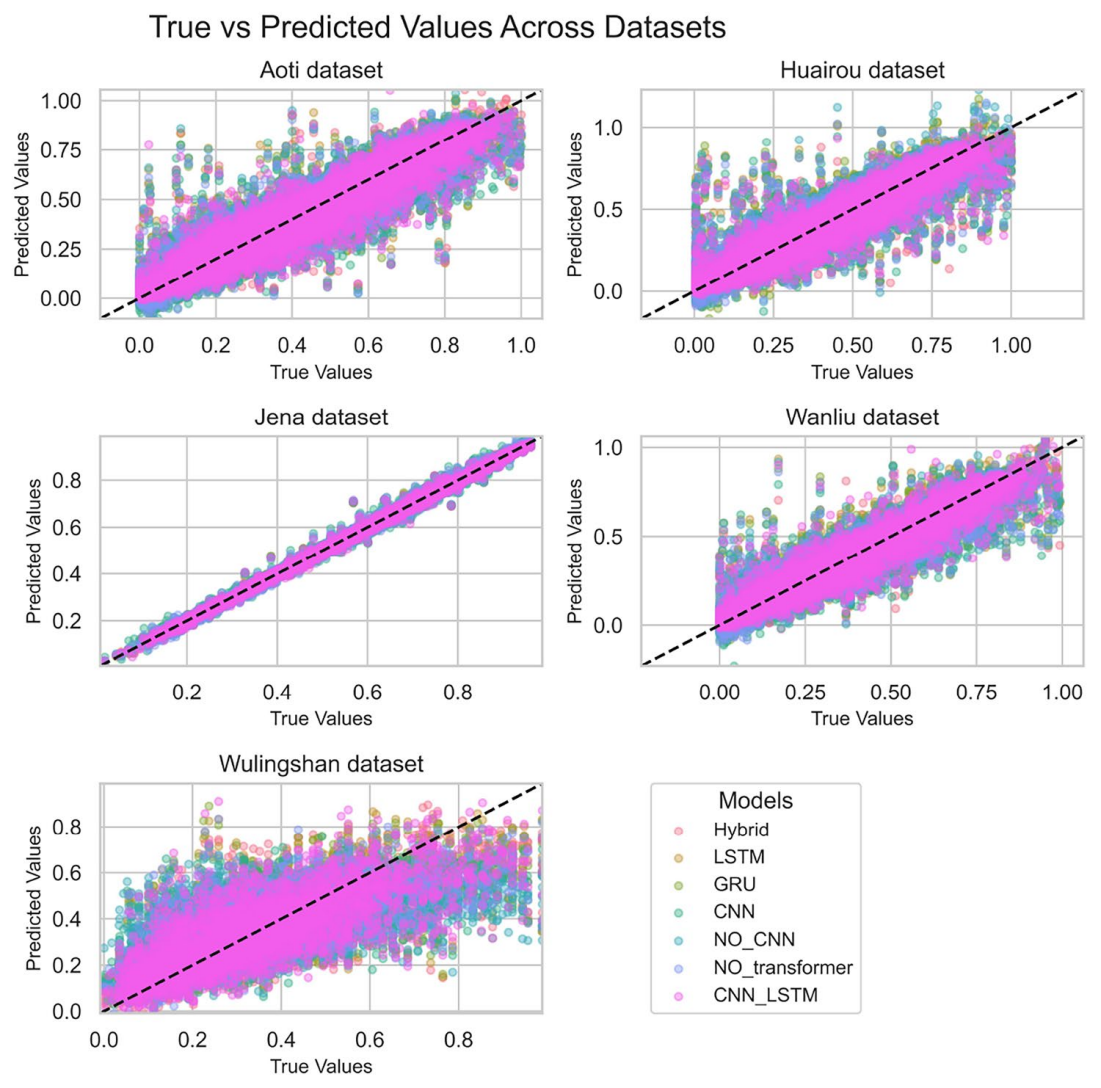
Comparison between true and predicted values across different datasets.

**Fig. 6:**
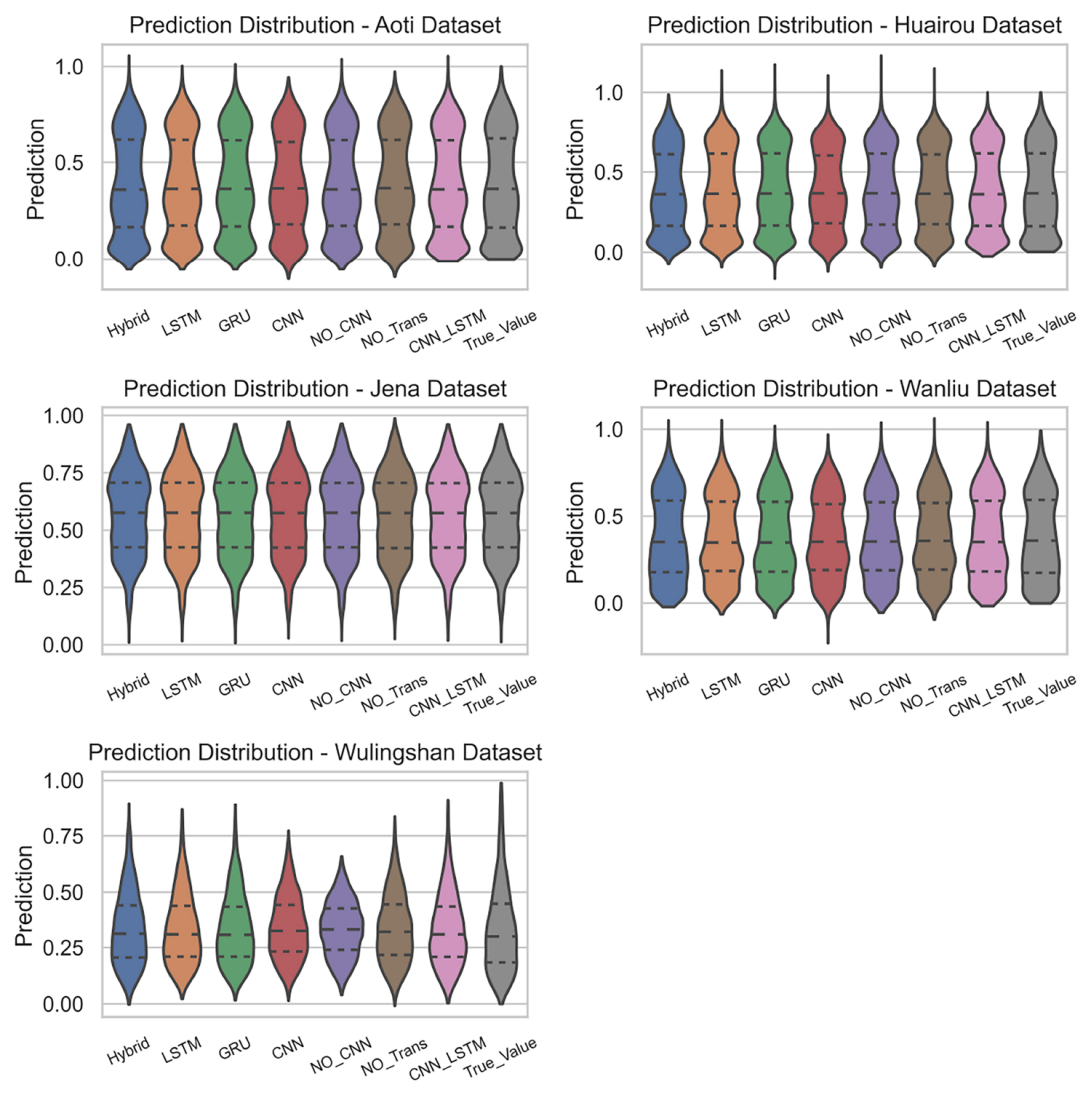
Prediction distributions of different models on various datasets (violin plots). Metrics: Predicted values for Target 1 and Target 2.

#### Analysis of the fit on different datasets

 As can be seen from the scatter plot in Figure 5, it shows the distribution of predicted values and true values of different models across various datasets. For a model with good robustness, its predicted values should cluster closely around the diagonal line of the true values, indicating small deviations between predicted and true values and stable performance across different datasets.

For all datasets (Aoti, Huairou, Jena, Wanliu, Wulingshan), the scatter points of the BG-Hybrid model mostly concentrate near the diagonal line. This indicates that the model can make relatively accurate predictions across different datasets, is capable of adapting to diverse data features and distributions, and demonstrates good robustness.

However, some other single models (such as LSTM, GRU, CNN, etc.) and models with structural limitations (NO_CNN, NO_Transformer) exhibit fluctuations in performance across different datasets, showing relatively weak robustness. The CNN-LSTM combined model has some improvement in robustness, but it is still less stable than the BG-Hybrid model.

This suggests that when dealing with diverse datasets, the BG-Hybrid model has an advantage in maintaining prediction accuracy and stability, and is more suitable for scenarios with high requirements for model robustness.

**Fig. 7:**
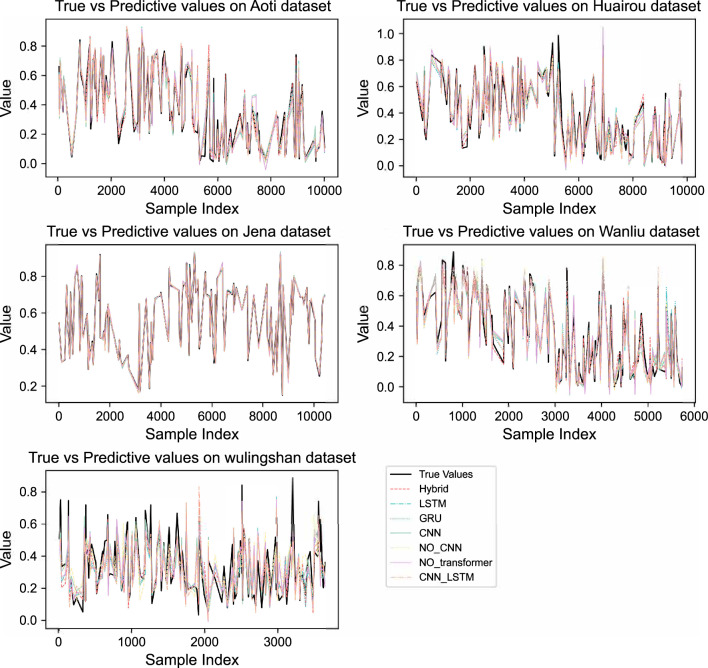
Fitting between the predicted value and the true value of different models.

#### Comparison of actual and predicted values on different datasets

Figure 7 shows the prediction results of various models on different datasets. It can be seen that the predictions of the BG-Hybrid model (pink lines) are relatively close to the true values (black lines). For example, in datasets such as the Aoti air quality dataset and Huairou air quality dataset, the trend of its curve has a high degree of overlap with that of the true-value curve, indicating that this model can well capture the data features on different datasets, with relatively high prediction accuracy, good stability, and generalization ability. Other models exhibit varying degrees of prediction deviation such as large prediction deviations and insufficient ability to capture data features on different datasets, and there are gaps compared with the BG-Hybrid model in terms of stability and generalization ability.

#### Distribution of predicted values on different datasets

Figure 6 shows the prediction distributions of multiple models on different datasets (Aoti, Huairou, Jena, Wanliu, Wulingshan) using violin plots. There are differences in the prediction distributions of various models across different datasets. For example, on the Aoti Dataset, the prediction distributions of most models are concentrated around higher predicted values; while on the Wulingshan Dataset, the prediction distributions of all models are more dispersed with lower peaks. This reflects that the data characteristics of different datasets (such as the degree of data fluctuation and distribution patterns) have a impact on the prediction performance of models. Some models perform well on certain datasets but may perform poorly on others.

**Fig. 8:**
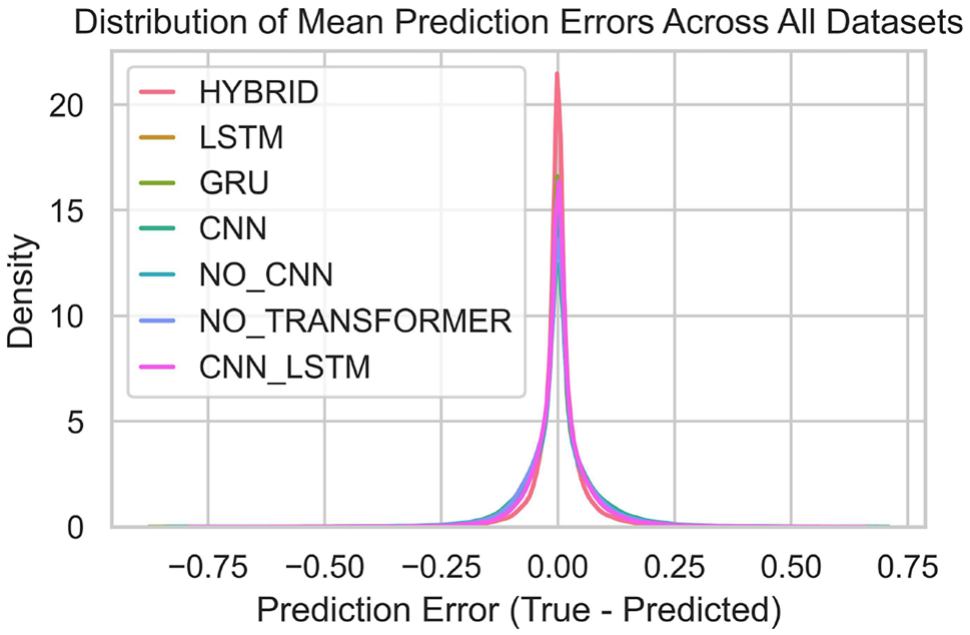
Distribution of mean Errors.

**Fig. 9:**
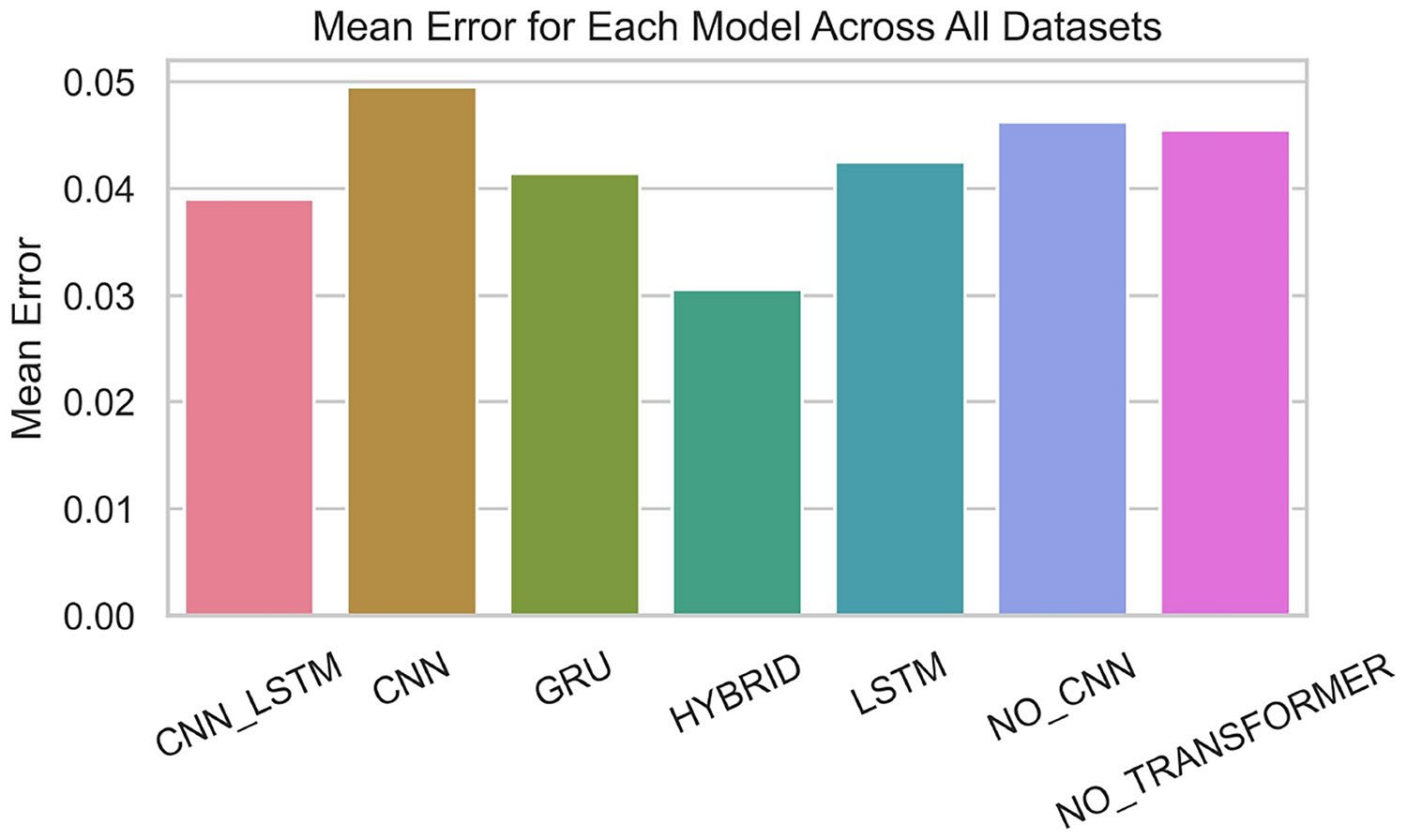
Mean error value.

In summary, the BG-Hybrid model has a more concentrated and stable prediction distribution across multiple datasets, with a clear advantage in prediction performance; other models have a larger degree of dispersion and poor stability in their prediction distributions across different datasets, showing weak adaptability and robustness to datasets. The characteristics of different datasets also significantly affect the prediction performance of models. Additionally, Figure 8 and Figure 9 show the prediction error distribution and values across all datasets, respectively.

## Discussion

### Model expressive ability

In our proposed BG-Hybrid model, the Transformer module and CNN module effectively capture the intrinsic multi-scale patterns within the data, achieving notable performance advantages. Experimental results demonstrate that removing individual modules from the BG-Hybrid model leads to a measurable performance degradation. For example, the absence of the self-attention mechanism of the Transformer leads to deficiencies in capturing long-range dependencies and integrating global information^32^. Additionally, when dealing with long text sequences, it is difficult to effectively associate semantic information over long distances. Furthermore, as shown in Table 3, removing the Bidirectional Gating Core (BGC) leads to varying degrees of degradation in MAE, RMSE, and R$\vphantom{0}^{2}$ metrics for both AQI and PM$\vphantom{0}_{2.5}$ prediction tasks. This confirms that the gating mechanism, which dynamically balances local and global features, is crucial for enhancing the model’s overall expressive ability.

### Limitations in model complexity and training efficiency

BG-Hybrid Model: Seeing a large number of parameters reaching 780,066 in Table 4, the model structure is complex. This makes the training process time-consuming and requires a large amount of computing resources, and has high requirements for hardware devices. At the same time, complex models are prone to overfitting. When the training data is limited, the model may over-learn the noise and special patterns in the training data and cannot generalize well to new data.

CNN_LSTM: The CNN_LSTM model exhibits reduced model complexity and fewer parameters compared to the BG-Hybrid model. However, training this architecture requires careful balancing between CNN’s convolutional operations and LSTM’s sequential processing, presenting non-trivial optimization challenges.

NO_CNN and NO_Transformer: Due to the lack of the characteristics of CNN and Transformer respectively, when dealing with complex tasks that require both local feature extraction and global information integration, they cannot flexibly handle various data features like some Hybrid models, and their versatility is insufficient.

**Table 3:** Comparison of performance with and without gating core.

Cross-Gating				NO Gating			
Target	MAE	RMSE	$\hbox {R}^{2}$	Target	MAE	RMSE	$\hbox {R}^{2}$
*AQI*	0.04781	0.06406	0.91395	*AQI*	0.05053	0.06648	0.90732
$PM_{2.5}$	0.05808	0.09118	0.85069	$PM_{2.5}$	0.05924	0.09230	0.84700

**Table 4:** Model parameters.

Models	Number of Parameters
BG-Hybrid	780,066
NO_Transformer	15,046
NO_CNN	69,254
CNN_LSTM	81,478
GRU	39,750
CNN	14,534
LSTM	52,358

### Weight analysis of bidirectional gating mechanism

To deeply analyze the dynamic feature fusion process of the BG-Hybrid model, we conducted research on the gating weights generated by the bidirectional gating mechanism ($\alpha$) for the CNN module and $\beta$ = 1 - $\alpha$ for the Transformer module). These weights can reflect the adaptive attention of the model to the local spatial features extracted by CNN and the global temporal dependencies captured by Transformer when processing Huairou data.

**Fig. 10:**
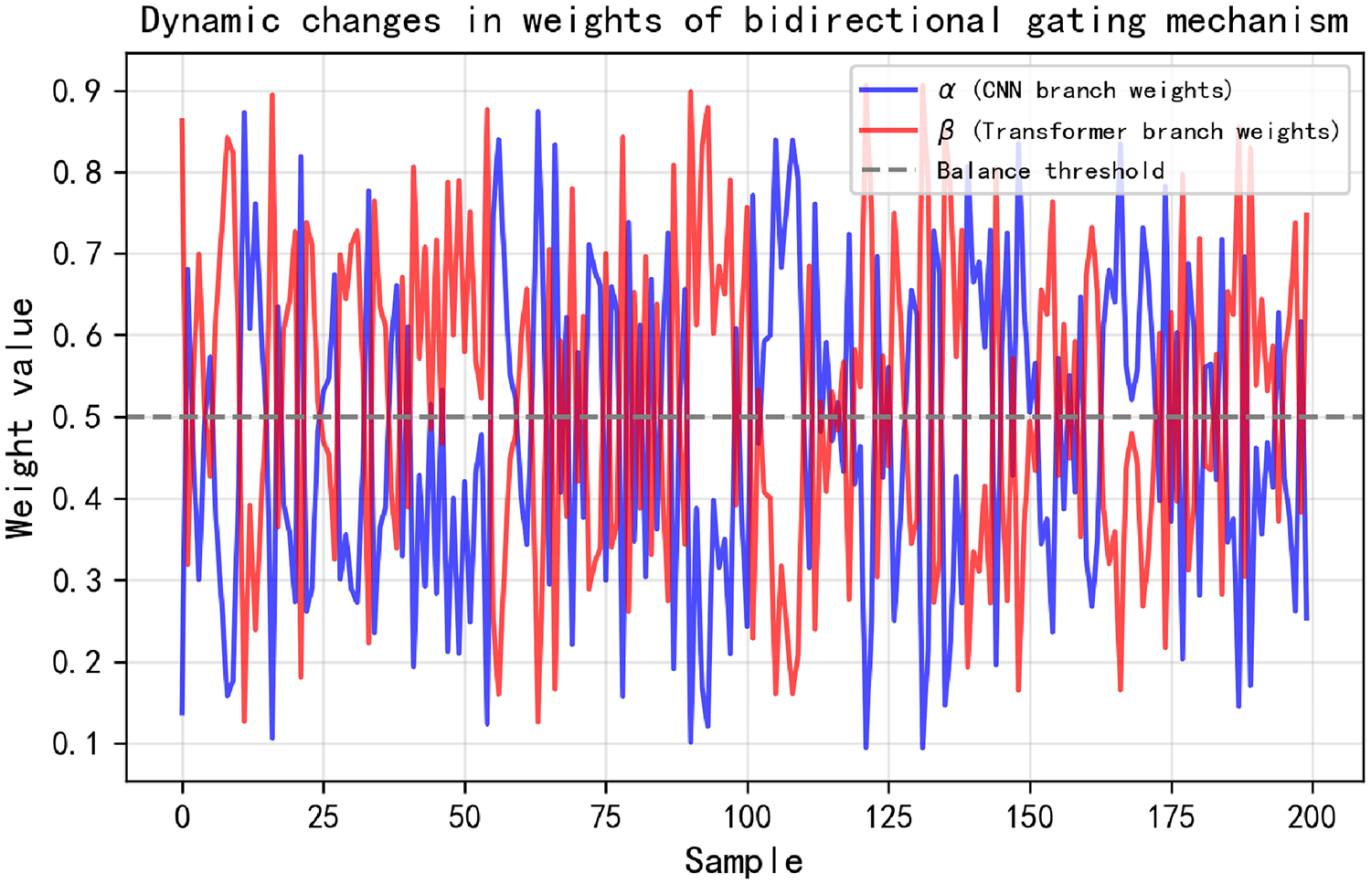
Dynamic changes of gating weights.

**Fig. 11:**
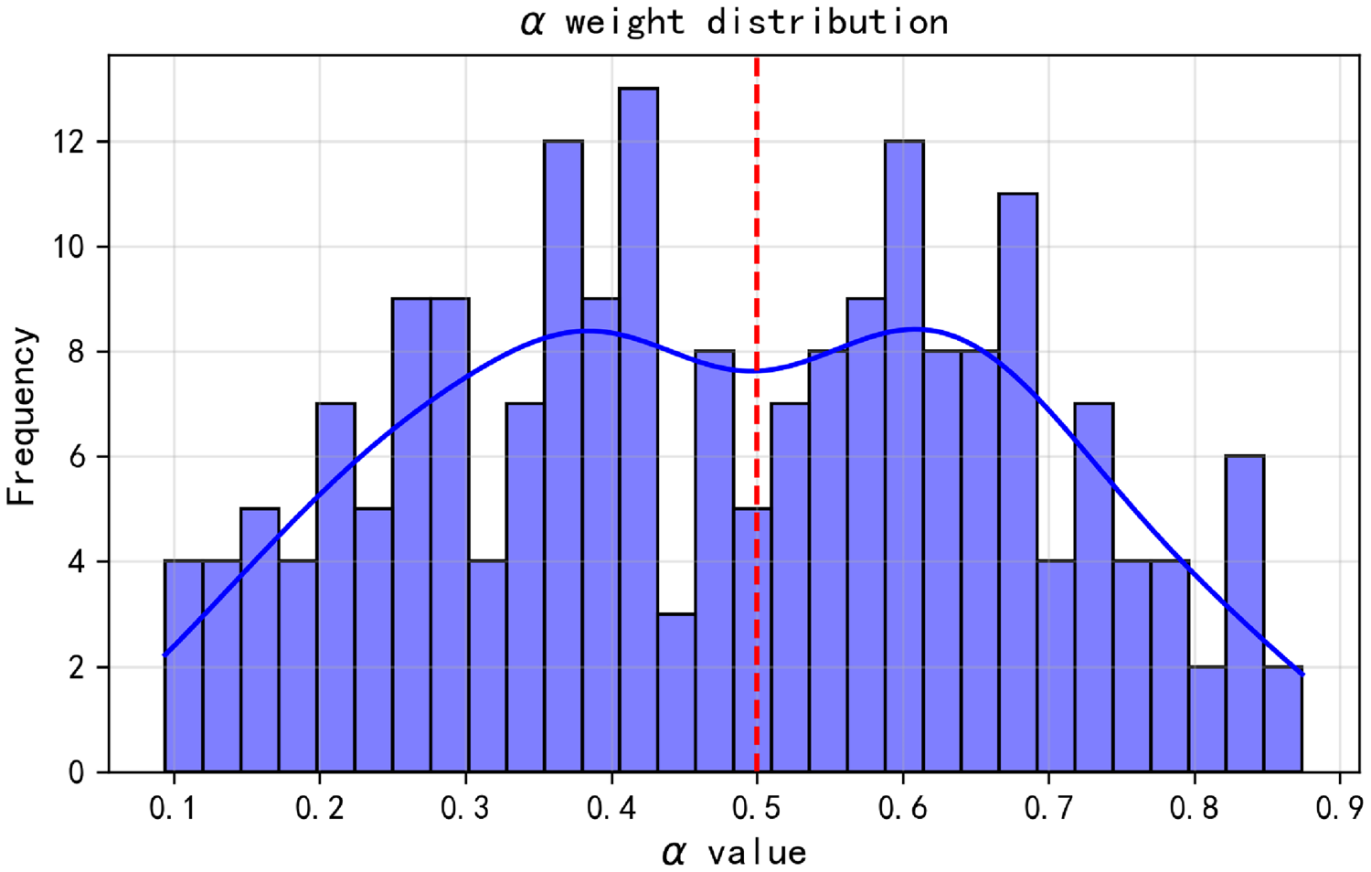
Distribution characteristics of $\alpha$ weights.

**Fig. re 12:**
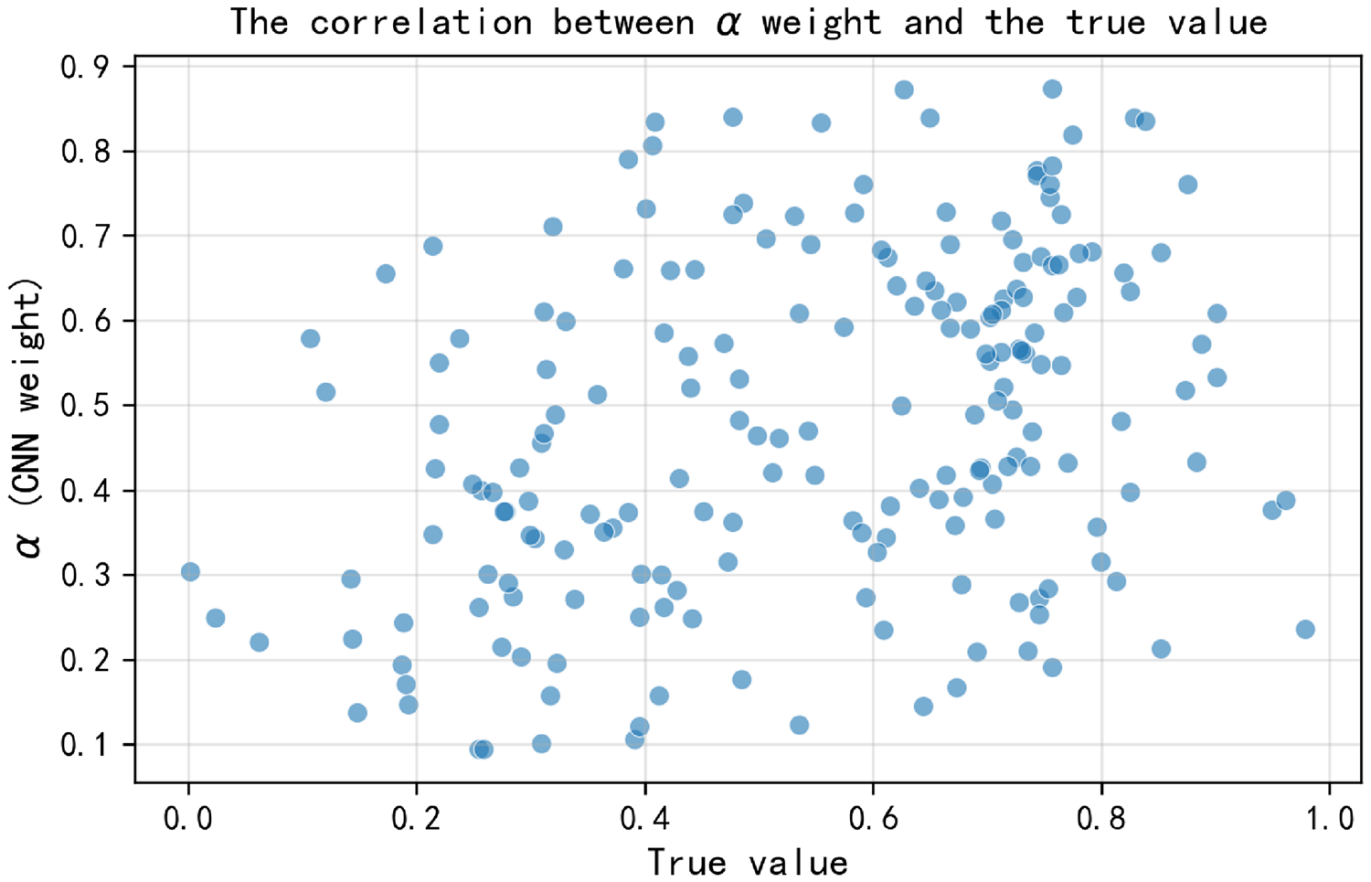
Correlation between $\alpha$ weights and true values.

### Dynamic changes of gating weights

Figure 10 shows the variation trends of $\alpha$ and $\beta$ weights with sample indices in the Huairou dataset. It can be seen from the figure that $\alpha$ and $\beta$ exhibit obvious dynamic fluctuation characteristics. In some stages of the data, the $\alpha$ value is relatively high (close to 0.8), which indicates that the model focuses more on using the local spatial features extracted by CNN to capture specific local pollution or environmental change patterns in Huairou data at this time; while in other stages, the $\beta$ value increases (that is, the $\alpha$ value decreases), indicating that the model instead relies on the global temporal modeling ability of Transformer to mine the long-term environmental change laws in Huairou data. This dynamic adjustment mechanism enables the model to flexibly balance the utilization of local and global features according to the actual characteristics of Huairou data, effectively improving the processing ability of complex environmental data.

### Distribution characteristics of alpha weights

Figure 11 presents the distribution of $\alpha$ weights in Huairou data. From the histogram and fitting curve, it can be seen that the distribution of $\alpha$ weights has certain characteristics. Its values are mainly concentrated in the interval of 0.3–0.7, and there is a relatively obvious concentration of $\alpha$ distribution around 0.5. This means that when processing Huairou data, the model will comprehensively consider the local spatial features of CNN and the global temporal features of Transformer in most cases. However, on the whole, the dependence on local spatial features (CNN module) is slightly stronger (because the $\alpha$ distribution is relatively more biased towards the higher value side). This distribution characteristic also reflects the characteristics of Huairou data from the side, that is, both local spatial features and global temporal features play important roles in the analysis and prediction of the model, and the model adaptively adjusts the degree of attention to them through the gating mechanism.

### Correlation between alpha weights and true values

Figure 12 is a scatter plot of $\alpha$ weights and the true values of Huairou data. By observing the distribution of scatter points, it can be found that there is a certain complex correlation between $\alpha$ weights and true values. In some intervals of true values, the $\alpha$ weights have a relatively concentrated distribution, indicating that when the data presents specific characteristics, the model will relatively stably adjust the degree of dependence on the local spatial features of CNN. However, on the whole, this correlation is not a simple linear relationship, which shows that when processing Huairou data, the model will dynamically and complexly balance the contributions of local and global features according to the environmental state reflected by the true values, so as to achieve more accurate analysis and prediction. This correlation analysis also verifies the effectiveness of the BGC, which can adaptively optimize the feature fusion method according to the actual situation of the data.

## Conclusion

We propose a BG-Hybrid model that improves the accuracy and robustness of air quality prediction via multi-branch feature learning and sequential modeling. By integrating the global temporal dependency modeling capability of Transformer, the local spatial feature extraction ability of CNN, and the dynamic temporal modeling strength of LSTM, the model alleviates the limitations of single-model approaches in capturing spatiotemporal dependencies. Experimental results demonstrate that the BG-Hybrid model achieves competitive performance compared to baseline models (CNN, LSTM, CNN-LSTM, etc.) across multiple air quality datasets, with the following key findings:

- Error Reduction: MAE and RMSE are reduced compared to single models (CNN/LSTM).
- Improved Goodness-of-Fit: The R^2^ score increases by an average of 3-12% shown in Table 2, indicating a better ability to capture underlying data patterns.
- Generalization Capability: The model achieves optimal performance for different prediction targets and maintains stable performance across diverse datasets.

Despite its promising performance, the BG-Hybrid model faces the following limitations:

- *High computational complexity*: With approximately 780k parameters, the model requires substantial training resources, which may limit its deployment in resource-constrained environments.
- *Performance variability across datasets*: While the model generally achieves strong results, its performance on certain datasets (e.g., Wulingshan) is not uniformly superior to all baselines, indicating that the optimal model choice may depend on specific data characteristics.
- *Limited interpretability*: The internal mechanisms of the bidirectional gating core and the contributions of individual modules are not fully explainable. Current work lacks integration of explainability techniques such as attention visualization or feature attribution analysis, which are important for practical decision-making support.
- *Data dependency*: The model’s effectiveness is evaluated on datasets with sufficient temporal continuity and completeness. Its performance on data with sparse observations or irregular sampling intervals remains unexplored.

Future research should focus on optimizing model efficiency through techniques such as pruning and quantization, while enhancing predictive capabilities by integrating multimodal data (e.g., satellite and radar imagery) and leveraging self-supervised learning with unlabeled air quality data to improve generalization in data-scarce scenarios. Additionally, developing interpretable visualization tools will be crucial for practical decision-making support.

The BG-Hybrid method provides an promising deep learning approach for air quality prediction, and its “global-local-temporal” collaborative modeling framework may be extended to other spatiotemporal prediction tasks, such as flood warnings and air quality prediction, warranting further investigation.

## Data Availability

The datasets used and/or analysed during the current study available from the corresponding author on reasonable request.
